# The Re-Emergence of Pediatric Pertussis: Insights from a Regional Romanian Hospital

**DOI:** 10.3390/antibiotics14070730

**Published:** 2025-07-21

**Authors:** Ioana Rosca, Alina Turenschi, Alexandru Dinulescu, Victoria Lichii

**Affiliations:** 1 Department of Neonatology, Faculty of Midwifery and Nursery, “Carol Davila” University of Medicine and Pharmacy, 050474 Bucharest, Romania; ioana.rosca@umfcd.ro; 2 Department of Obstetrics and Gynecology, Clinical Hospital of Obstetrics and Gynecology “Prof. Dr. Panait Sârbu”, 3–5 Giulesti Street, 060251 Bucharest, Romania; 3 Pediatric Hospital Ploiesti, 100326 Ploiesti, Romania; victoria.boldescu@rez.umfcd.ro; 4 Department of Pediatrics, Faculty of Medicine, “Carol Davila” University of Medicine and Pharmacy, 050474 Bucharest, Romania; 5 Department of Pediatrics, Emergency Hospital for Children “Grigore Alexandrescu”, 011743 Bucharest, Romania

**Keywords:** pertussis, vaccine hesitancy, pediatric infectious diseases, maternal immunization, Romania, whooping cough

## Abstract

Introduction: Pertussis, a vaccine-preventable disease caused by Bordetella pertussis, is resurging globally due to declining immunization rates. This study explores the clinical and epidemiological features of pediatric pertussis cases in a regional Romanian hospital amid growing vaccine hesitancy. Methods: We conducted a retrospective cohort study on 99 children diagnosed with pertussis and admitted to Ploiești Pediatric Hospital between January 2024 and January 2025. Demographic, clinical, laboratory, and radiological data were analyzed using SPSS 25.0. Results: The median age was 11 months (IQR 4–25), with 12.1% under two months, and ineligible for the first DTaP dose. Notably, 72.7% of children were unvaccinated; 59.4% had missed scheduled doses. None of the mothers received the DTaP vaccination during pregnancy. Most cases (55.6%) had bilaterally accentuated interstitial patterns on chest X-ray, significantly associated with vaccination status (*p* = 0.019). The leukocyte count was higher in children with alveolar infiltrates (*p* = 0.028), and as the number of vaccine doses increased, the leukocyte count tended to slightly decrease (*p* = 0.022, R = −0.229). PCR confirmation was obtained after a mean of 2.2 days, with 12.1% of cases confirmed post-discharge. Azithromycin was used in 74.7% of cases, with good tolerability. Conclusions: Low pediatric and maternal vaccine uptake was a major contributor to pertussis resurgence in this cohort. Radiological severity correlated with vaccination status, suggesting that vaccination may confer protection not only against infection but also against severe pulmonary involvement. These findings support urgent public health efforts to restore vaccine confidence and coverage, particularly among vulnerable infant populations and expectant mothers.

## 1. Introduction

Pertussis (whooping cough) is a highly contagious respiratory disease caused by Bordetella pertussis with high morbidity and mortality rates in infants and especially in unvaccinated children [1,2,3,4]. Infection with pertussis progresses through three stages. The first is the catarrhal stage (the stage when the patient is the most infectious), similar to other upper respiratory tract infections, with fever, rhinorrhea, and conjunctivitis, followed by the paroxysmal stage, with repeated rapid coughing that is followed by the characteristic “whoop”; these paroxysm episodes can be followed by vomiting, cyanosis, or syncope. The last stage is the convalescent one, which is characterized by a residual cough that can last several months [4,5,6].

Despite the availability of an effective vaccine since the mid-20th century, pertussis continues to re-emerge in cyclical outbreaks worldwide, particularly in regions with declining vaccine coverage; for example, the incidence rate (IR) raised in Europe from 3.4 per million in 2021 to 104.4 per million, and there are countries from other regions such as China or South Africa who reported a resurgence after the COVID-19 pandemic as well [4,7,8,9,10]. In recent years, several European countries have reported a concerning increase in pediatric pertussis cases, underlining the vulnerability of certain population groups [7,9,10,11]. The classic presentation typically occurs as a primary infection in unvaccinated children <10 years of age, but it also may occur in vaccinated children and adults [12,13,14,15].

The vaccination schedule in Romania includes DTaP (diphtheria, tetanus, acellular pertussis) vaccination during infancy (2, 4, 11 months) and later in childhood (6 and 14 years), and it is also recommended in pregnancy after 16 gestational weeks [16,17]. However, vaccination coverage has shown signs of erosion in recent years; European countries have recorded a decrease in the third dose of DTaP from 95% in 2019 to 93% in 2024, with 95% being considered the threshold value to achieve herd immunity [18,19,20]. Multiple factors contribute to this trend: insufficient access to healthcare in rural areas, inconsistent public health messaging, and growing vaccine hesitancy fueled by misinformation.

The COVID-19 pandemic, while centered on SARS-CoV-2 vaccination, has exacerbated public distrust in immunization efforts overall. In Romania, vaccine skepticism, fueled by social media misinformation and polarized public discourse, has not only affected the acceptance of new vaccines but also undermined confidence in long-established ones. This lack of trust has led to reduced uptake of routine childhood vaccines, placing infants, especially those too young to be fully immunized, at renewed risk for preventable diseases such as pertussis [19,21,22,23,24,25].

In this context, this study aimed to analyze the clinical and epidemiological features of pediatric pertussis cases in a regional Romanian hospital and to explore associations between vaccination status and disease severity.

## 2. Results

### 2.1. Patient Demographics

**Figure 1:**
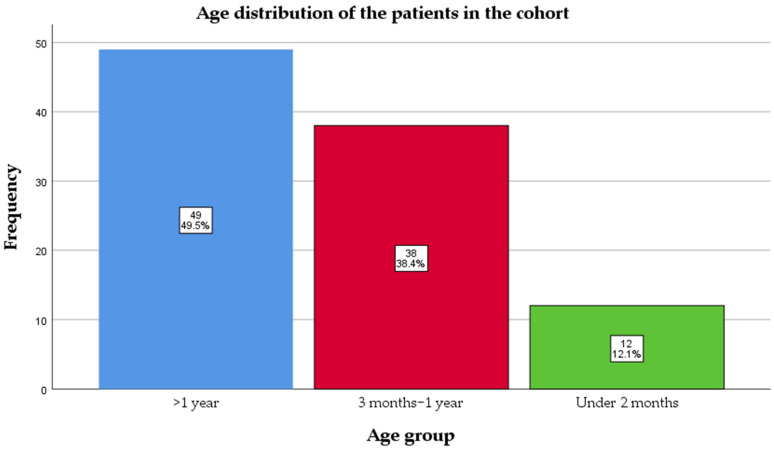
Age distribution of the patients in the cohort.

Ninety-nine pediatric patients with PCR-confirmed pertussis were included in this retrospective cohort (January 2024–January 2025). The median age at admission was 11 (4–25) months. Almost half of them, 50 (50.5%), were infants, and 12 (12.1%) were under 2 months old, meaning they had not reached the age for the first dose of vaccine (Figure 1).

**Figure 2:**
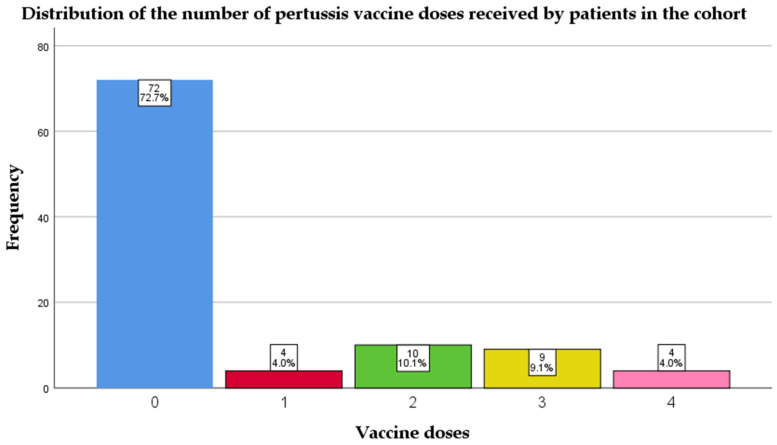
Distribution of the number of pertussis vaccine doses received by patients in the cohort.

Vaccination coverage against pertussis was remarkably low in this cohort; 72 (72.7%) did not receive any vaccine dose (Figure 2).

**Figure 3:**
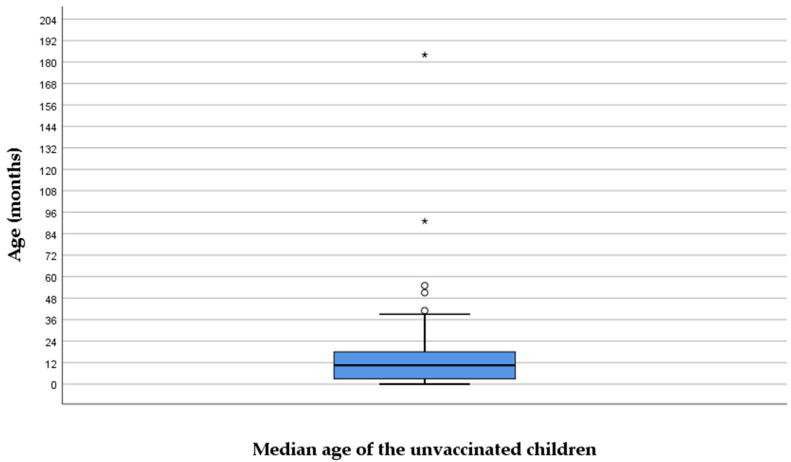
Median age of the unvaccinated children. The interior bars indicate the medians while the whiskers extend to the maximum and minimum of the data; ◦ = outlier; * = far outlier.

Sixty patients did not receive any vaccine dose even though they reached the vaccination age. The median age of those unvaccinated was 14.5 (6–23) months (Figure 3), the oldest patient being 15 years old. None of the mothers received a tetanus–diphtheria–acellular pertussis booster during pregnancy.

The median time of disease for hospital admission was 5 (3–10) days. There is a statistically significant relationship (*p* = 0.046) with a weak positive correlation (R = 0.171) between a patient’s age and the number of days from the onset of their illness to their hospital admission. Older children tended to be admitted to the hospital slightly later, after getting sick, compared to younger children. However, their age was only a minor factor in this delay.

### 2.2. Laboratory Findings

The CRP level at admission had a median of 0.5 (0.1–3.6) mg/dL. We found a statistically significant (*p* = 0.034), weak positive correlation (R = 0.213) between a patient’s age and their CRP level, meaning that there is a real, but weak, tendency for older children to have slightly higher CRP levels compared to younger children. There was no correlation between the CRP level and days of onset (*p* = 0.158, R = −0.143) or vaccine doses (*p* = 0.158; R = 0.144).

The leukocytes at admission had a median count of 16,000 (9000–23,100) × 10^9^/L. There was no correlation between leukocytes and age (*p* = 0.178; R = 0.027) and days of onset (*p* = 0.918; R = −0.046). A Spearman’s rho correlation analysis was conducted to assess the relationship between leukocyte count and C-reactive protein. The results revealed a weak but statistically significant positive correlation between leukocyte count and CRP levels (R = 0.248, *p* = 0.013). This suggests that, as leukocyte counts increase, CRP levels also tend to rise, although the strength of this correlation is modest. There is a weak correlation between leukocyte count and vaccine dose (*p* = 0.022, R = −0.229), meaning that as the number of vaccine doses increased, the leukocyte count tended to slightly decrease.

The average turnaround time for PCR results was 2.2 ± 1.4 days, reflecting a short delay between sampling and laboratory confirmation. While this is a relatively rapid diagnostic method, in practice, it meant that definitive results became available roughly 48 h after the child’s admission, on average. In 88 of 99 cases, pertussis was confirmed by PCR while the patient was still hospitalized, guiding further management. However, in 12 patients (12.1%), the PCR result became available only after the child had already been discharged from the hospital. These were typically patients with shorter hospital stays or those who improved quickly; their pertussis diagnosis was essentially confirmed retrospectively after they left. The total time of diagnosis (days of onset + PCR turnaround time) was 7 (5–13) days.

### 2.3. Radiology

**Figure 4:**
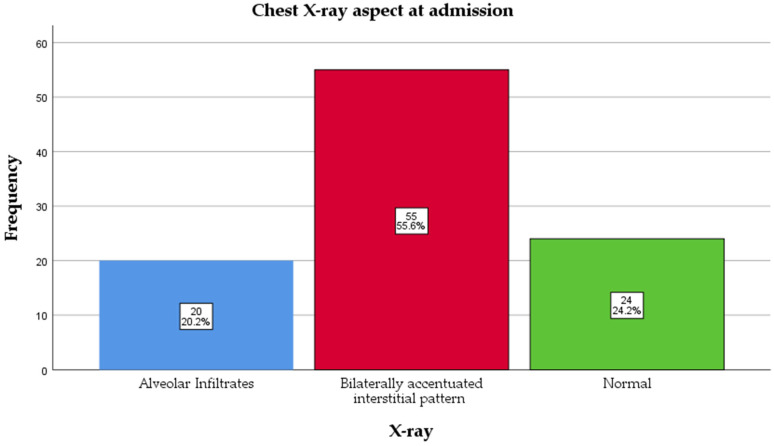
Chest X-ray aspect at admission.

All patients underwent a chest X-ray at admission, and assessments were read by a radiologist blinded to vaccination status. The majority had a bilaterally accentuated interstitial pattern, 55 (55.6%), followed by a normal aspect, 24 (24.2%), and alveolar infiltrates, 20 (20.2%) (Figure 4). There was no association between X-ray and age (*p* = 0.315) or days of onset (*p* = 0.042, but *p* > 0.05 was found between groups after the Bonferroni adjustment for multiple comparisons); for CRP, *p* = 0.331. The group with alveolar infiltrates had a higher count of leukocytes than the normal X-ray group, 23,350 (13,250–35,000) vs. 13,000 (11,000–16,750) × 10^9^/L (*p* = 0.028).

The distribution of radiological findings varied across vaccination status.

Among unvaccinated patients (0 doses), the majority (59.7%) showed bilaterally accentuated interstitial patterns, while 23.6% had alveolar infiltrates, and 16.7% had normal X-rays. In contrast, patients with two doses showed a predominance of normal X-rays (70%), with only 10% having alveolar infiltrates and 20% showing interstitial patterns (*p* = 0.019). In those with three or four doses, bilaterally accentuated interstitial patterns were the most common finding (66.7% and 50%, respectively), but these categories had a small sample size (Table 1).

### 2.4. Hospitalization

The hospitalization time had a median of 4 (2–7) days and was not associated with age (*p* = 0.064), X-ray findings (*p* = 0.776), or vaccination doses (*p* = 0.364).

### 2.5. Treatment

**Figure 5:**
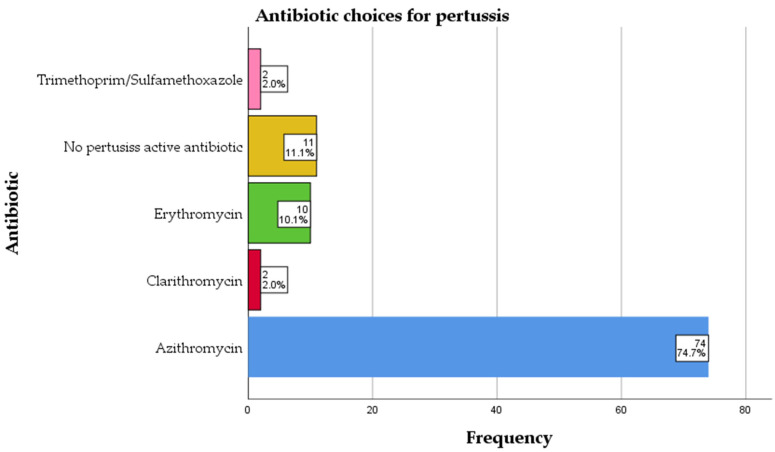
Antibiotic choices for pertussis.

Nearly all patients received appropriate antibiotic therapy targeted against Bordetella pertussis during their hospitalization. A pertussis-active macrolide or antibiotic was initiated in 88 out of 99 cases (88.9%), usually at the time of clinical diagnosis (before PCR confirmation) or immediately after a positive PCR result. Azithromycin was the first-line treatment in the vast majority of these cases: it was used in about 74.7%. A smaller proportion of children received erythromycin (10.1% of cases), typically in situations such as drug availability issues or clinician preference. Very few patients were treated with alternative agents: clarithromycin was used in 2% of cases, and trimethoprim–sulfamethoxazole in another 2%. These choices reflect second-line options for pertussis, for example, in cases of macrolide intolerance or contraindication. Antibiotic treatment was well tolerated, and no significant drug-related adverse events were reported in the cohort. Notably, 11 patients (11.1%) did not receive any specific anti-pertussis antibiotic during their hospital stay. In most of these cases, this was because the patient’s hospital stay was very brief and the confirmatory PCR result indicating pertussis became available only after discharge (as described above). In such instances, either the clinical suspicion of pertussis was initially low or the child improved rapidly, and thus no macrolide was started. For those patients, appropriate follow-up was arranged, and prophylactic antibiotics were offered to close contacts as per public health guidelines. The distribution of antibiotic choices is summarized in Figure 5.

## 3. Discussion

This retrospective cohort study highlights several critical aspects of pertussis epidemiology in a Romanian pediatric population, particularly concerning vaccination coverage, clinical presentation, and diagnostic approach. A key finding of concern was the remarkably low vaccination rate—nearly three-quarters (72.7%) of the patients had not received any dose of pertussis vaccine, and over half (59.4%) were unvaccinated despite having reached the eligible age. Mihai et al. (2025) report a similar trend in their study, where they analyzed pertussis infection in 38 children from a Romanian county (Constanta); in their study, 31 (81.5%) were unvaccinated [5]. Both of these studies confirm the national trend of a decrease in vaccination, seen also in other outbreaks, such as measles, rotavirus, or influenza [26,27,28,29,30,31].

These findings suggest a concerning decline in routine childhood vaccination uptake, which may increase the risk of outbreaks of vaccine-preventable diseases such as pertussis, particularly among infants who are not yet fully immunized. This trend reflects the broader impact of the COVID-19 pandemic, which has disrupted healthcare services and intensified public mistrust in vaccination through misinformation and polarized discourse [32,33]. The high percentage of unvaccinated people in our study also confirms the protective efficiency of the vaccine against acquiring the disease [4,10,34].

The age distribution of cases also emphasizes the vulnerability of infants too young to be vaccinated; 12.1% of the cohort were under 2 months of age and therefore ineligible for the first DTaP dose. This underscores the importance of maternal immunization during pregnancy, a well-documented protective strategy that was notably absent in this cohort [35,36,37]. None of the mothers had received a tetanus–diphtheria–acellular pertussis (DTaP) booster during pregnancy, indicating missed opportunities for passive immunity.

Our study found a weak correlation between leukocyte count and vaccine dose (*p* = 0.022, R = −0.229), meaning that as the number of vaccine doses increases, the leukocyte count tends to slightly decrease. To our knowledge, no previous studies have explored this correlation, but it is known that a very high level of leukocytes is associated with poor outcomes [38,39].

Over half of the children showed bilaterally accentuated interstitial changes (55.6%), consistent with the known pulmonary involvement in pertussis [4,5,40]. Importantly, radiological severity appeared to correlate with vaccination status; children with two or more vaccine doses were more likely to have normal chest X-rays, while unvaccinated children more often exhibited interstitial or alveolar patterns. To our knowledge, no previous study has reported an association between chest X-ray findings and vaccination status in pediatric pertussis cases.

Despite prompt hospitalization, delays in confirmatory PCR testing led to a diagnostic lag in 12% of cases, with some results returning only after discharge. Although the mean turnaround time was relatively short (2.2 days), these delays hindered early confirmation, potentially affecting the timely initiation of appropriate empirical antibiotic therapy and delaying implementation of necessary isolation precautions. The median time to diagnosis was 7 days (IQR: 5–13), underscoring the importance of recognizing pertussis primarily based on clinical presentation [41,42].

Azithromycin was the predominant antibiotic used, in line with international guidelines, and was well tolerated. The low use of clarithromycin or alternative agents reflects clinical preference and national prescribing trends. The absence of adverse events is reassuring and supports the safety of early macrolide treatment, even prior to microbiological confirmation [43,44]. However, it is important to acknowledge that antimicrobial resistance against azithromycin is increasing globally, raising public health concerns; in fact, the World Health Organization has classified azithromycin as an antibiotic with a high risk of antimicrobial resistance [45,46,47].

To address declining vaccine confidence, especially in the wake of the COVID-19 pandemic, public health efforts should prioritize concrete strategies such as community-based education campaigns, enhanced training for healthcare providers on vaccine communication, and targeted maternal immunization programs aimed at protecting newborns through passive immunity.

## 4. Materials and Methods

We conducted a retrospective cohort study using electronic medical records and diagnostic codes. Informed consent was obtained from all subject families involved in the study. We included children (0–17 years old) admitted to Ploiești Pediatric Hospital who were confirmed to have Bordetella pertussis infection in the period of January 2024–January 2025. The exclusion criteria were a lack of data and the refusal of the parents to include the children in the study. The patients were confirmed through multiplex PCR testing for respiratory pathogens, which is a faster method in our laboratory than serology. Ninety-nine patients met the inclusion criteria, and no patient was excluded.

The data was collected by the authors in Microsoft Office Excel 2013 from the electronic medical records of the hospital and double-checked. The data collected included patient demographics (age, sex, area of provenance), clinical data (duration of disease until hospital admission), laboratory tests (PCR, CRP, leukocyte count), and imaging (chest X-ray).

The data was analyzed and illustrated using IBM SPSS Statistics version 25. Quantitative variables were tested for normal distribution using the Shapiro–Wilk test, and because the data did not meet normality assumptions as assessed by the Shapiro–Wilk test, they were written as medians with interquartile ranges (IQRs). Quantitative variables were tested between independent groups using Mann–Whitney U tests. The Kruskal–Wallis test was used to determine significant differences between two or more groups of an independent variable. Fisher’s exact test was used to determine the nonrandom associations between categorical variables, with the Bonferroni method used for correction. The Spearman coefficient was used to search for correlations between non-normal distributed quantitative variables.

## 5. Conclusions

Low vaccine uptake remains a major driver of pertussis resurgence in Romania. More than 70% of children in this cohort were unvaccinated, and over half had missed scheduled doses despite being eligible. Infants too young to be vaccinated remain highly vulnerable, highlighting the critical need to improve maternal DTaP vaccination coverage. The findings underscore the importance of strengthening public health education and vaccine advocacy in Romania, particularly in light of increased vaccine hesitancy in the post-COVID-19 era. Targeted public health interventions are essential to strengthen routine childhood immunization and maternal vaccination to prevent future outbreaks of pertussis.

## Figures and Tables

**Table 1:** Chest X-ray findings stratified by the number of pertussis vaccine doses received.

X-Ray Finding	0 Doses	1 Dose	2 Doses	3 Doses	4 Doses	Total
Alveolar Infiltrates	17 (23.6%; 95% CI: 13.8–33.4%)	0 (0.0%; 95% CI: 0.0–0.0%)	1 (10.0%; 95% CI: −8.6–28.6%)	2 (22.2%; 95% CI: −4.9–49.4%)	0 (0.0%; 95% CI: 0.0–0.0%)	20 (20.2%)
Bilaterally Accentuated Interstitial Patterns	43 (59.7%; 95% CI: 48.4–71.1%)	2 (50.0%; 95% CI: 1.0–99.0%)	2 (20.0%; 95% CI: −4.8–44.8%)	6 (66.7%; 95% CI: 35.9–97.5%)	2 (50.0%; 95% CI: 1.0–99.0%)	55 (55.6%)
Normal	12 (16.7%; 95% CI: 8.1–25.3%)	2 (50.0%)	7 (70.0%; 95% CI: 41.6–98.4%)	1 (11.1%; 95% CI: −9.4–31.6%)	2 (50.0%; 95% CI: 1.0–99.0%)	24 (24.2%)
Total (*n*)	72 (100%)	4 (100%)	10 (100%)	9 (100%)	4 (100%)	99 (100%)

## Data Availability

Data are contained within the article.

## Abbreviations

The following abbreviations are used in this manuscript:

CRP	C-reactive protein
PCR	Polymerase chain reaction
DTaP	Diphtheria, tetanus, acellular pertussis
